# Supplementary material for: Shotgun Metagenomic Sequencing to Assess Cyanobacterial Community Composition following Coagulation of Cyanobacterial Blooms
Source: Toxins (Basel). 2022 Oct 7;14(10):688. doi: 10.3390/toxins14100688 (PMC9607033; doi:10.3390/toxins14100688)
Supplement: Supplementary file 1 [file toxins-14-00688-s001.zip › toxins-1939413-supplementary.pdf]

Supplementary Material

Shotgun Metagenomic Sequencing to Assess Cyanobacterial Community Composition following Coagulation of Cyanobacterial Blooms

Kim Thien Nguyen Le [1,*], Juan Francisco Guerra Maldonado [1], Eyerusalem Goitom [2], Hana Trigui [3], Yves Terrat [4], Thanh-Luan Nguyen [1], Barry Husk [5], B. Jesse Shapiro [4,6,7], Sébastien Sauvé [8], Michèle Prévost [1] and Sarah Dorner [1]

[1] Department of Civil, Geological and Mining Engineering, Polytechnique de Montréal, Montréal, QC H3C 3A7, Canada
[2] Department of Geography and Environmental Studies, Toronto Metropolitan University, Toronto, ON M5B 2K3, Canada
[3] Institut National de Santé Publique de Quéquec, Montréal, QC H2P 1E2, Canada
[4] Department of Biological Sciences, University of Montréal, Montréal, QC H2V 0B3, Canada
[5] BlueLeaf Inc., 310 Chapleau Street, Drummondville, QC J2B 5E9, Canada
[6] McGill Genome Centre, McGill University, Montréal, QC H3A 0G1, Canada
[7] Department of Microbiology and Immunology, McGill University, Montréal, QC H3A 2B4, Canada
[8] Department of Chemistry, University of Montréal, Montréal, QC H3C 3J7, Canada
* Correspondence: thien-kim.le-nguyen@polymtl.ca

Table S1. Removal effectiveness (%) of taxonomic cell counts of individual cyanobacterial genus after 48 hours (Mean±Standard deviation) in Missisquoi Bay (MB) and Petit Lac St. François (PLSF). Adapted from the author of [1].

Event	Treat	**Total cell counts**	*Aphanizomenon*	*Aphanocapsa*	*Aphanothece*	*Chroococcus*	*Coelospharium*	*Dolichospermum*	*Merismopedia*	*Microcystis*	*Pseudanabaena*
**MB September 10-12, 2018**	20 mgFe/L	99.96±0.04	97.84±3.05	100.0±0.00	98.30±0.21	100.0±0.00	NA	99.88±0.04	100.0±0.00	99.97±0.01	99.97±2.81
	35 mgFe/L	99.94±0.04	97.69±1.12	100.0±0.00	97.27±0.11	100.0±0.00	NA	99.81±0.05	100.0±0.00	99.65±0.05	97.75±0.79
**MB September 24-26, 2018**	20 mgFe/L	71.91±5.38	68.55±1.94	89.27±6.55	64.38±2.83	NA	NA	75.22±7.48	100.0±0.00	86.92±2.14	72.69±3.93
	35 mgFe/L	96.39±1.29	80.58±7.53	76.56±2.31	69.09±1.63	NA	NA	98.51±1.19	100.0±0.00	93.98±2.61	71.59±8.54
**MB August 13-15, 2019**	20 mgFe/L	94.27±1.67	93.91±1.64	NA	87.73±6.22	NA	100.0±0.00	97.17±3.14	NA	86.55±1.62	NA
	35 mgFe/L	99.35±0.11	99.26±0.15	NA	96.51±1.44	NA	100.0±0.00	99.97±0.01	NA	100.0±0.00	NA
**PLSF June 26-28, 2019**	20 mgFe/L	85.22±6.43	77.17±8.16	NA	-37.5±1.67	NA	35.11±5.01	84.66±4.07	NA	92.66±8.64	NA
	35 mgFe/L	98.99±1.06	98.66±1.44	NA	88.50±4.58	NA	100.0±0.00	99.03±0.95	NA	95.82±4.36	NA
**PLSF July 24-26, 2019**	20 mgFe/L	51.98±6.21	76.76±4.51	NA	-13.5±1.24	100.0±0.00	19.36±1.14	66.57±4.71	77.5±0.28	66.99±0.95	NA
	35 mgFe/L	99.11±0.11	97.93±0.28	NA	92.65±0.96	100.0±0.00	100.0±0.00	100.0±0.00	100.0±0.00	100.0±0.00	NA
**PLSF August 05-07, 2019**	20 mgFe/L	78.21±6.72	72.22±6.32	NA	79.47±4.98	100.0±0.00	75.86±4.33	77.97±5.04	92.14±3.59	96.51±5.39	NA
	35 mgFe/L	99.72±0.65	98.22±0.65	NA	93.46±2.34	NA	100.0±0.00	100.0±0.00	100.0±0.00	99.97±0.01	NA

Removal effectiveness percentage (%) = $\frac{T0 - T48}{T0} \times 100$. NA: no value.

Table S2. Removal effectiveness (%) of taxonomic cell counts of individual cyanobacterial genus after 48 hours on August 08-10, 2018, in Missisquoi Bay (Mean ± Standard deviation).

Event	Treat	Total cell counts	*Aphanocapsa*	*Aphanothece*	*Dolichospermum*	*Microcystis*
August 08-10, 2018	20 mgFe/L	50.01±0.04	100.0±0.00	53.36±0.21	77.88±0.04	99.97±0.01
	35 mgFe/L	96.94±0.04	100.0±0.00	67.27±0.11	97.81±0.05	

Removal effectiveness percentage (%) = $\frac{T0 - T48}{T0} \times 100$. NA: no value

Table S3. Mean ± standard deviation value for environmental conditions of the sampled water in the control mesocosms on August 08-10, 2018, in Missisquoi Bay (n=2).

Parameters	August 08 2018	August 10 2018
**Chlorophyll-*a* (RFU)**	20.01 ± 2.45	-
**Phycocyanin (RFU)**	155.16 ± 2.96	-
**pH**	8.61 ± 0.07	-
**DO (mg/L)**	9.11 ± 0.21	-
**Temp (°C)**	26.70 ± 0.21	-
**TOC (mg C/L)**	2387.5 ± 45.96	185.51 ± 4.01
**DOC (mg C/L)**	38.41 ± 0.27	36.10 ± 1.46
**TN (mg N/L)**	14.44 ± 0.83	14.85 ± 0.69
**TP (µg P/L)**	3155.01 ± 67.45	1769.02 ± 89.39
**DN (mg N/L)**	0.10 ± 0.001	5.46 ± 0.57
**DP (µg P/L)**	328.22 ± 2.07	168.58 ± 5.79

- No data.

Table S4. Environmental conditions of lake water samples in control mesocosms (n = 6) at T0 (Mean ± standard deviation). Adapted from author of [1].

Parameters	Missisquoi Bay			Petit Lac St. François		
	**(A) 10 September 2018**	**(B) 24 September 2018**	**(C) 13 August 2019**	**(a) 26 June 2019**	**(b) 24 July 2019**	**(c) 05 August 2019**
Total cell counts (cells/mL)	998,183 ± 35,034	547,325 ± 16,578	17,408,158 ± 138,898	6,033,197 ± 316,425	109,193 ± 3578	235,723 ± 5986
Chlorophyll-*a* (RFU)	-	-	62.22 ± 1.03	3.97 ± 0.04	5.14 ± 0.06	6.27 ± 0.08
Phycocyanin (RFU)	-	-	93.43 ± 0.52	16.51 ± 1.54	1.77 ± 0.04	4.83 ± 0.13
pH	6.5 ± 0.08	6.4 ± 0.07	8.05 ± 0.24	8.08 ± 0.07	7.84 ± 0.03	8.01 ± 0.01
TDS (mg/L)	101 ± 0.00	100 ± 0.00	98.00 ± 0.00	122.50 ± 0.71	116.00 ± 0.00	115.00 ± 0.00
Temp (°C)	21.8 ± 0.01	18.7 ± 0.12	26.89 ± 0.21	25.96 ± 0.49	25.14 ± 0.12	24.43 ± 0.24
TOC (mg C/L)	15.22 ± 0.25	5.55 ± 0.07	885.00 ± 33.34	175.00 ± 9.50	11.10 ± 0.38	10.76 ± 0.13
DOC (mg C/L)	7.50 ± 0.05	5.00 ± 0.00	19.34 ± 1.67	9.80 ± 0.11	9.83 ± 0.13	11.34 ± 1.58
TN (mg N/L)	5.55 ± 0.65	2.75 ± 0.08	7.84 ± 2.95	12.85 ± 0.57	1.11 ± 0.05	1.29 ± 0.008
TP (µg P/L)	360.51 ± 0.01	292.11 ± 13.28	4336.92 ± 74.65	723.55 ± 24.97	110.38 ± 1.89	131.78 ± 7.71
DN (mg N/L)	0.45 ± 0.004	0.52 ± 0.009	2.66 ± 0.12	0.73 ± 0.08	0.60 ± 0.02	0.50 ± 0.02
DP (µg P/L)	17.05 ± 0.07	17.99 ± 0.45	302.17 ± 12.98	139.06 ± 3.35	23.92 ± 4.91	42.86 ± 1.13

- : No data.

Table S5. Environmental conditions of lake water samples in control mesocosms at T48 (Mean ± standard deviation). Adapted from author of [1].

Parameters	Missisquoi Bay			Petit-Lac-St-François		
	Event A September 12 2018	Event B September 26 2018	Event C August 15 2019	Event a June 28 2019	Event b July 26 2019	Event c August 07 2019
**Chlorophyll-*a* (RFU)**	-	-	64.72 ± 0.59	3.37 ± 0.32	5.63 ± 0.16	7.88 ± 0.17
**Phycocyanin (RFU)**	-	-	169.21 ± 0.34	16.42 ± 0.15	0.87 ± 0.03	6.78 ± 0.16
**pH**	7.47 ± 0.07	-	6.33 ± 0.09	9.93 ± 0.06	8.36 ± 0.07	7.37 ± 0.15
**TDS (mg/L)**	105 ± 0.00	-	140.0 ± 4.24	151.0 ± 5.65	118.5 ± 0.71	121.0 ± 0.00
**Temp (°C)**	22.7 ± 0.17	-	22.09 ± 0.17	27.81 ± 0.41	25.37 ± 0.34	25.25 ± 0.02
**TOC (mg C/L)**	19.97 ± 0.24	5.46 ± 0.00	700.0 ± 23.19	11.39 ± 0.23	10.57 ± 0.21	10.19 ± 0.44
**DOC (mg C/L)**	12.17 ± 0.39	5.08 ± 0.05	73.48 ± 1.45	9.83 ± 0.06	10.07 ± 0.83	9.81 ± 0.07
**TN (mg N/L)**	5.46 ± 0.69	1.68 ± 0.01	6.84 ± 1.78	11.21 ± 1.07	1.01 ± 0.15	1.28 ± 0.05
**TP (µg P/L)**	320.92 ± 4.48	177.49 ± 21.03	2074.60 ± 20.22	603.01 ± 3.17	72.08 ± 5.32	89.29 ± 4.81
**DN (mg N/L)**	2.02 ± 0.001	0.48 ± 0.009	1.56 ± 0.11	0.91 ± 0.25	0.58 ± 0.00	0.63 ± 0.03
**DP (µg P/L)**	40.47 ± 1.17	15.43 ± 0.25	215.01 ± 11.61	108.06 ± 2.22	16.46 ± 1.16	21.81 ± 2.13

- : No data.

Table S6. Environmental conditions of lake water samples in mesocosms with dose of 20 mgFe/L at T48 (Mean ± standard deviation).

Parameters	Missisquoi Bay			Petit-Lac-St-François		
	Event A September 12 2018	Event B September 26 2018	Event C August 15 2019	Event a June 28 2019	Event b July 26 2019	Event c August 07 2019
**Chlorophyll-*a* (RFU)**	-	-	1.04 ± 0.002	0.08 ± 0.00	1.18 ± 0.01	0.82 ± 0.07
**Phycocyanin (RFU)**	-	-	33.69 ± 5.21	0.49 ± 0.05	0.54 ± 0.00	1.03 ± 0.05
**pH**	4.9 ± 0.04	-	5.08 ± 1.01	6.09 ± 0.01	7.70 ± 0.19	5.66 ± 0.02
**TDS (mg/L)**	138 ± 0.00	-	152.00 ± 0.00	130.00 ± 0.00	139.00 ± 0.00	146.00 ± 0.00
**Temp (°C)**	21.8 ± 0.01	-	22.95 ± 0.04	23.92 ± 0.02	25.21 ± 0.01	25.03 ± 0.01
**TOC (mg C/L)**	4.52 ± 0.15	2.22 ± 0.09	23.76 ± 4.52	17.56 ± 2.71	3.81 ± 0.51	3.69 ± 0.11
**DOC (mg C/L)**	3.21 ± 0.03	2.39 ± 0.03	22.62 ± 1.92	15.10 ± 2.87	3.63 ± 0.24	3.44 ± 0.01
**TN (mg N/L)**	0.67 ± 0.01	1.29 ± 0.01	4.16 ± 1.01	3.71 ± 0.48	0.55 ± 0.007	0.35 ± 0.009
**TP (µg P/L)**	8.47 ± 0.35	126.02 ± 14.54	167.73 ± 6.34	130.02 ± 14.49	20.12 ± 1.15	14.73 ± 0.71
**DN (mg N/L)**	0.64 ± 0.001	0.39 ± 0.01	3.28 ± 0.29	1.28 ± 0.04	0.36 ± 0.02	0.23 ± 0.001
**DP (µg P/L)**	5.51 ± 0.02	7.14 ± 0.14	60.08 ± 12.79	19.34 ± 1.44	6.72 ± 1.56	3.56 ± 0.21

Table S7. Environmental conditions of lake water samples in mesocosms with dose of 35 mgFe/L at T48 (Mean ± standard deviation).

Parameters	Missisquoi Bay			Petit-Lac-St-François		
	Event A September 12 2018	Event B September 26 2018	Event C August 15 2019	Event a June 28 2019	Event b July 26 2019	Event c August 07 2019
**Chlorophyll-*a* (RFU)**	-	-	1.05 ± 0.002	0.01 ± 0.00	0.09 ± 0.007	0.01 ± 0.00
**Phycocyanin (RFU)**	-	-	29.69 ± 1.13	0.05 ± 0.00	0.11 ± 0.00	0.015 ± 0.007
**pH**	3.95 ± 0.08	-	4.01 ± 0.13	4.47 ± 0.15	4.1 ± 0.02	4.01 ± 0.05
**TDS (mg/L)**	198 ± 0.00	-	221.5 ± 9.81	285.01 ± 7.12	228.00 ± 0.00	296.50 ± 2.12
**Temp (°C)**	21.05 ± 0.01	-	22.83 ± 0.12	27.73 ± 0.33	25.26 ± 0.03	24.69 ± 0.33
**TOC (mg C/L)**	4.01 ± 0.06	2.18 ± 0.09	22.02 ± 0.41	5.68 ± 0.34	2.27 ± 0.31	1.68 ± 0.01
**DOC (mg C/L)**	3.71 ± 0.01	2.41 ± 0.03	21.07 ± 0.91	7.31 ± 0.10	1.87 ± 0.21	1.87 ± 0.10
**TN (mg N/L)**	0.67 ± 0.01	1.07 ± 0.02	4.06 ± 0.15	0.95 ± 0.01	0.28 ± 0.007	0.29 ± 0.007
**TP (µg P/L)**	8.83 ± 0.56	109.97 ± 23.21	146.44 ± 7.81	29.29 ± 0.73	6.58 ± 1.71	4.86 ± 0.82
**DN (mg N/L)**	0.63 ± 0.001	0.41 ± 0.007	2.93 ± 0.08	0.72 ± 0.04	0.25 ± 0.001	0.27 ± 0.00
**DP (µg P/L)**	7.11 ± 0.04	6.92 ± 0.04	64.71 ± 4.23	17.99 ± 2.02	4.49 ± 0.11	4.01 ± 0.19

Table S8. Pairwise Kruskal-Wallis test, showing differences in changing of the species richness and Shannon indices at genus level between control mesocosms and mesocosms with dose of 20 mgFe/L, control mesocosms and mesocosms with dose of 35 mgFe/L, mesocosms with dose of 20 mgFe/L and 35 mgFe/L after 48 hours in Missisquoi Bay and Petit Lac St. François (p-value< 0.05).

		df	*chi-squared*	*p*-value
Missisquoi Bay	***Richness***	1	1.218	0.264
	Shannon	1	0.561	0.452
Petit Lac St. François	***Richness***	1	1.334	0.248
	Shannon	1	0.044	0.833

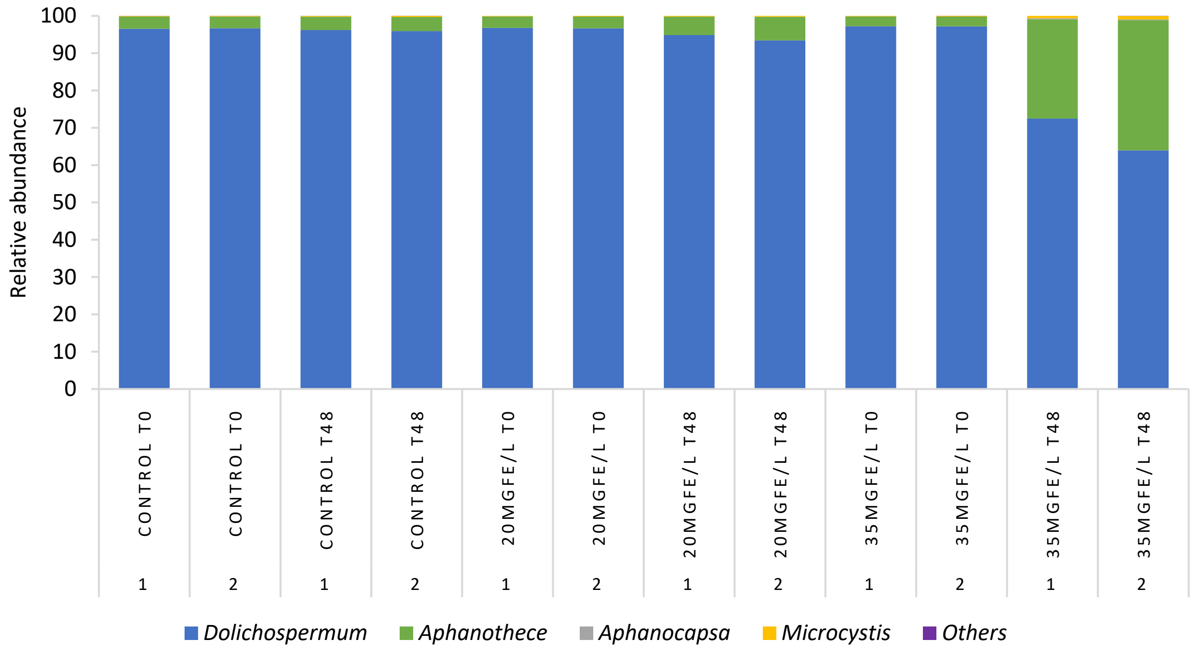

Figure S1. Cyanobacterial relative abundance at genus level in the control, 20 mgFe/L and 35 mgFe/L mesocosms in Missisquoi Bay in August 08 (T0) and August 10 (T48), 2018.

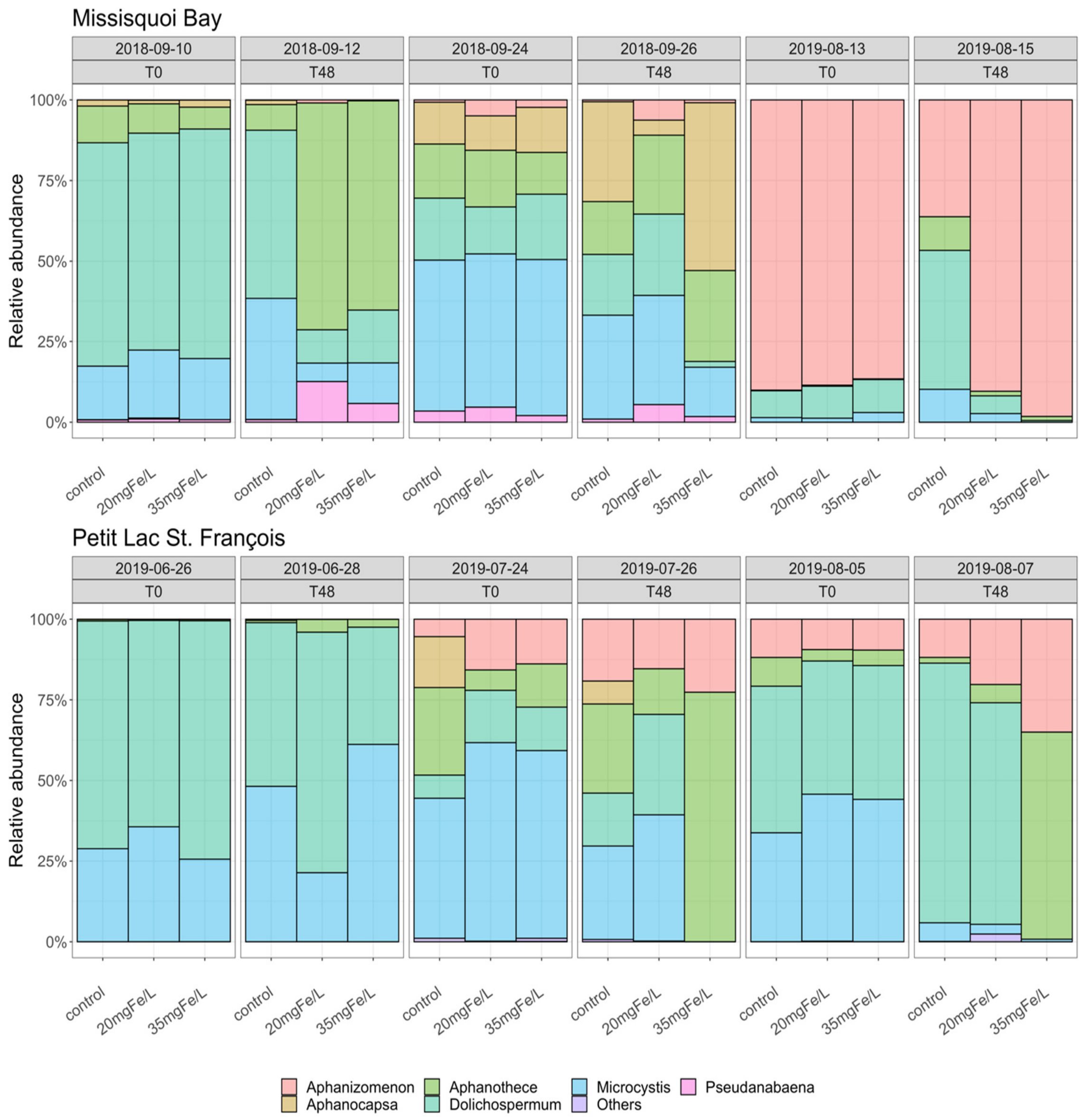

Figure S2. Cyanobacterial relative abundance at genus level in the control, 20 mgFe/L and 35 mgFe/L mesocosms in Missisquoi Bay and Petit Lac St. François. Adapted from [1].

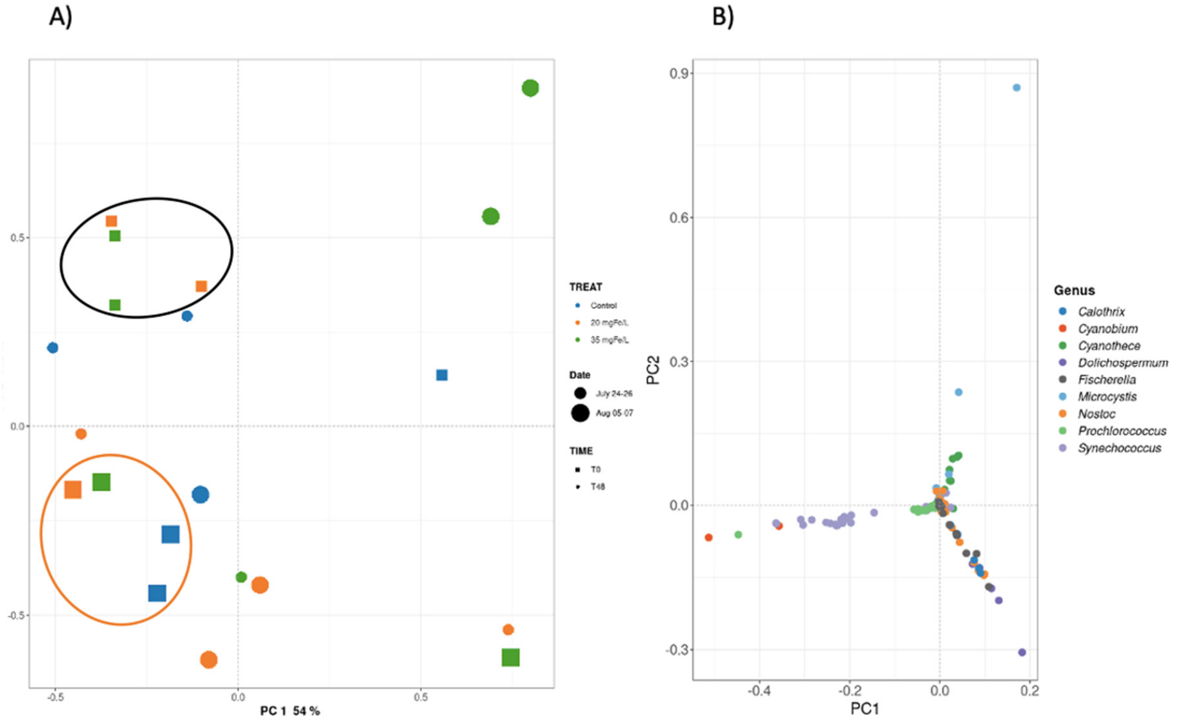

Figure S3. Principal components analysis (PCA) of the normalized relative abundance of cyanobacteria community composition in control, 20 mgFe/L and 35 mgFe/L mesocosms with respect to genus abundance in Petit Lac St. François. (a) PCA analysis of cyanobacterial community following coagulation; (b) Data are plotted following the genus-level classification.

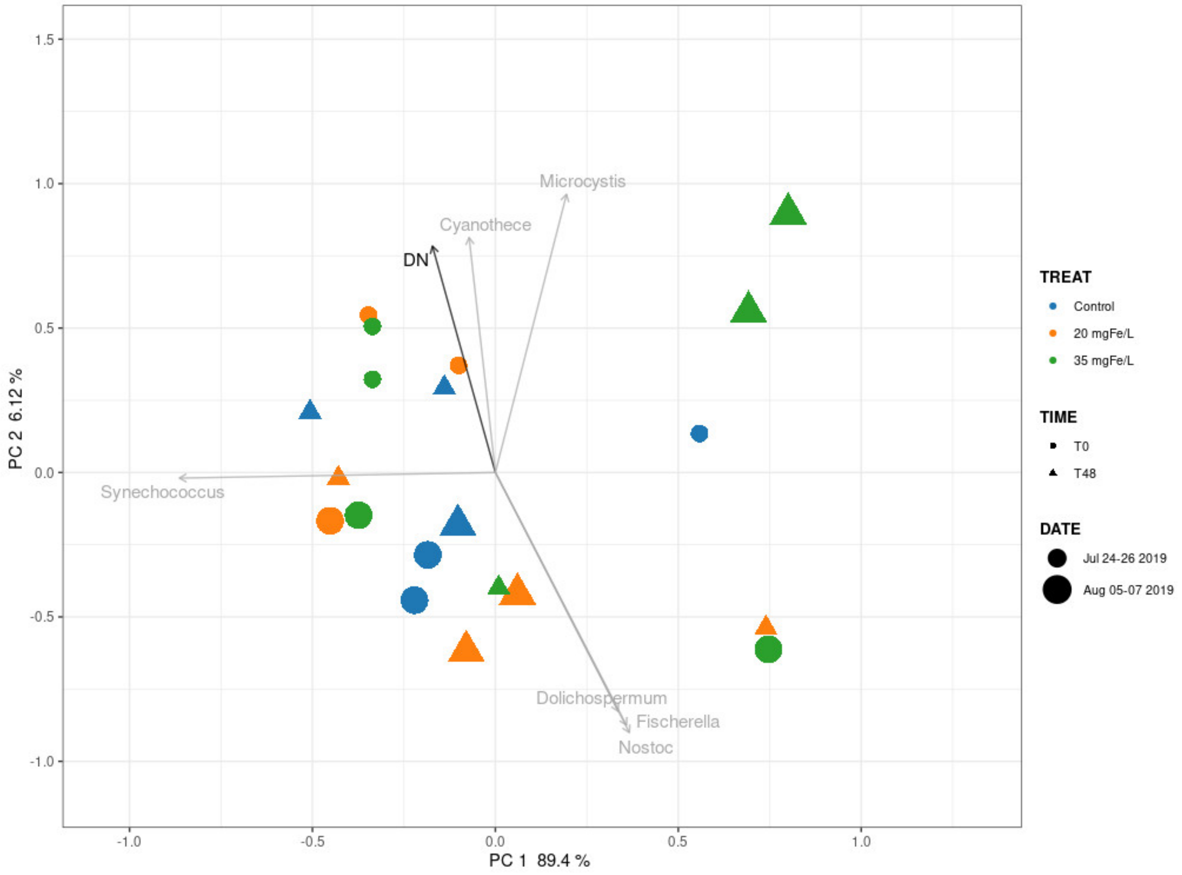

Figure S4. Principal components analysis (PCA) of the normalized relative abundance of cyanobacteria community composition in control, 20 mgFe/L and 35 mgFe/L mesocosms with respect to genus abundance in Petit Lac St. François (PLSF). (a) PCA analysis of cyanobacterial community following coagulation; (b) Data are plotted following the genus-level classification.

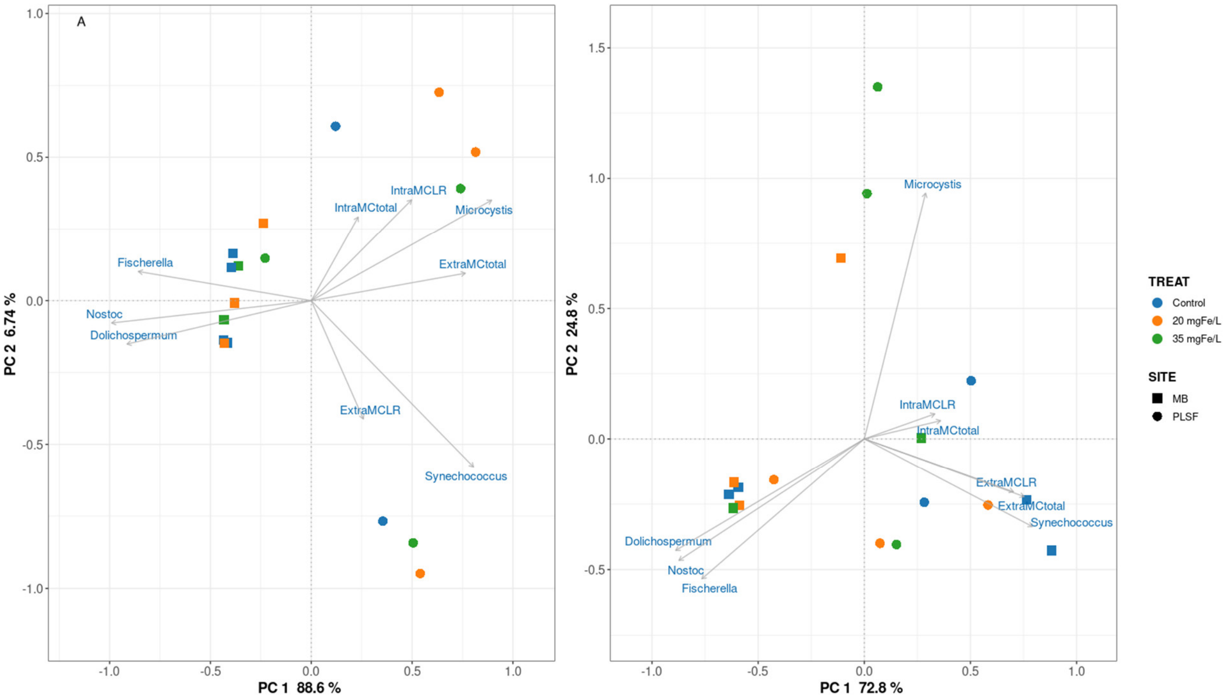

Figure S5. Principal components analysis (PCA) of the normalized relative abundance of cyanobacteria community composition in control, 20 mgFe/L and 35 mgFe/L mesocosms with respect to intra- and extracellular total microcystins, MC-LR in Missisquoi Bay and Petit Lac St. François at T0 (left panel) and T48 (right panel).

References

1. Le, K.T.; Goitom, E.; Trigui, H.; Sauvé, S.; Prévost, M.; Dorner, S. The Effects of Ferric Sulfate (Fe2(SO4)3) on the Removal of Cyanobacteria and Cyanotoxins: A Mesocosm Experiment. *Toxins* **2021**, *13*, 753. https://doi.org/10.3390/toxins13110753.
